# Ecosystem fragmentation drives increased diet variation in an endemic livebearing fish of the Bahamas

**DOI:** 10.1002/ece3.1140

**Published:** 2014-07-31

**Authors:** Márcio S Araújo, R Brian Langerhans, Sean T Giery, Craig A Layman

**Affiliations:** 1 Departamento de Ecologia, Instituto de Biociências, Universidade Estadual Paulista “Julio de Mesquita Filho”Rio Claro, Sao Paulo, 13506-900, Brazil; 2 Department of Biological Sciences and W.M. Keck Center for Behavioral Biology, North Carolina State UniversityBox 7617, Raleigh, North Carolina, 27695-7617; 3 Marine Sciences Program, Department of Biological Sciences, Florida International University3000 NE 151st St, North Miami, Florida, 33181

**Keywords:** Bahamas mosquitofish, food webs, individual specialization, niche variation, predation, RNA/DNA ratios, stable isotopes

## Abstract

One consequence of human-driven habitat degradation in general, and habitat fragmentation in particular, is loss of biodiversity. An often-underappreciated aspect of habitat fragmentation relates to changes in the ecology of species that persist in altered habitats. In Bahamian wetlands, ecosystem fragmentation causes disruption of hydrological connectivity between inland fragmented wetlands and adjacent marine areas, with the consequent loss of marine piscivores from fragmented sections. We took advantage of this environmental gradient to investigate effects of ecosystem fragmentation on patterns of resource use in the livebearing fish *Gambusia hubbsi* (Family Poeciliidae), using both population- and individual-level perspectives. We show that fragmentation-induced release from predation led to increased *G. hubbsi* population densities, which consequently led to lower mean growth rates, likely as a result of higher intraspecific competition for food. This was accompanied by a broadening of dietary niches via increased interindividual diet variation, suggesting a negative effect of predation and a positive effect of intraspecific competition on the degree of diet variation in natural populations. Our results therefore indicate that habitat fragmentation can greatly impact the ecology of resilient populations, with potentially important ecological and evolutionary implications.

## Introduction

Human-induced habitat degradation often results in population declines and loss of biodiversity (Saunders et al. 1991; Fahrig 2003; Foley et al. 2005; Fischer and Lindenmayer 2007). Top predators are especially susceptible, with predator declines associated with altered community structure and ecosystem function. Predator declines also lead to fundamental shifts in the ecology of individuals that persist in degraded ecosystems. This individual-level perspective has received much less attention than other aspects of ecological change in degraded ecosystems (Estes et al. 2011).

Previous work has demonstrated major impacts of predatory release on the evolution of life-history traits, secondary sexual traits, and functional morphological traits of prey species (Langerhans et al. 2007; Reznick et al. 2008; Walsh and Reznick 2009; Riesch et al. 2013; Martin et al. 2014). Resource use by prey species may also vary across predation gradients via behavioral changes in prey caused by the presence (or absence) of predators. For example, in the presence of a predator, prey individuals may concentrate in a safer, homogeneous microhabitat, potentially constraining their food niche (Eklöv and Svanbäck 2006). Alternatively, predators can reduce prey density through direct consumption, so that predation release should result in higher densities and increased intraspecific competition among prey (Bassar et al. 2010). In turn, niche width is expected to expand as competition becomes stronger and preferred resources become scarce (optimal foraging theory [OFT]; Stephens and Krebs 1986). Predation release, therefore, is expected to cause niche expansion in prey, either because prey individuals can occupy previously unexplored microhabitats or experience increased intraspecific competition for food.

Regardless of the mechanism underlying niche expansion (behavioral or density dependent), it can be achieved via increased individual niches (OFT) or increased interindividual variation – also known in the literature as “individual specialization” (Bolnick et al. 2003; Araújo et al. 2011) or “niche variation” (Van Valen 1965; Bolnick et al. 2007). Increased interindividual variation is expected when differences in phenotype or experience among individuals cause them to differ in their rank preferences for resources, so that population niche expansion may result in higher niche variation (Svanbäck and Bolnick 2005). Niche variation has been demonstrated to have important ecological and evolutionary implications (Bolnick et al. 2003, 2011), and a growing literature has investigated how resource gradients and ecological interactions such as intra- and interspecific competition affect the degree of niche variation in natural settings. Available empirical examples suggest a positive effect of resource diversity and intraspecific competition and a negative effect of interspecific competition on the degree of niche variation (reviewed in Araújo et al. 2011), but there is only a single study of the effect of predation on niche variation (Eklöv and Svanbäck 2006).

Ecosystem fragmentation represents a pervasive anthropogenic impact across the planet and provides a major source of declines in top predators (Turner 1996; Vitousek et al. 1997; Dirzo and Raven 2003). Although terrestrial ecosystems have dominated the focus of discussions on fragmentation (Harrison and Bruna 1999; Debinski and Holt 2000; Fahrig 2003), aquatic ecosystem fragmentation is also common and can similarly affect biodiversity (Nilsson et al. 2005; Pringle 2006). For example, in Caribbean coastal systems, disruption of hydrological connectivity between wetlands and adjacent marine areas causes the severe reduction or extirpation of marine piscivores (Layman et al. 2004, 2007; Valentine-Rose et al. 2007a,b; Rypel and Layman 2008). This loss of top predators may have important impacts on the ecological attributes of the resilient species that persist in disturbed areas.

**Figure 1:**
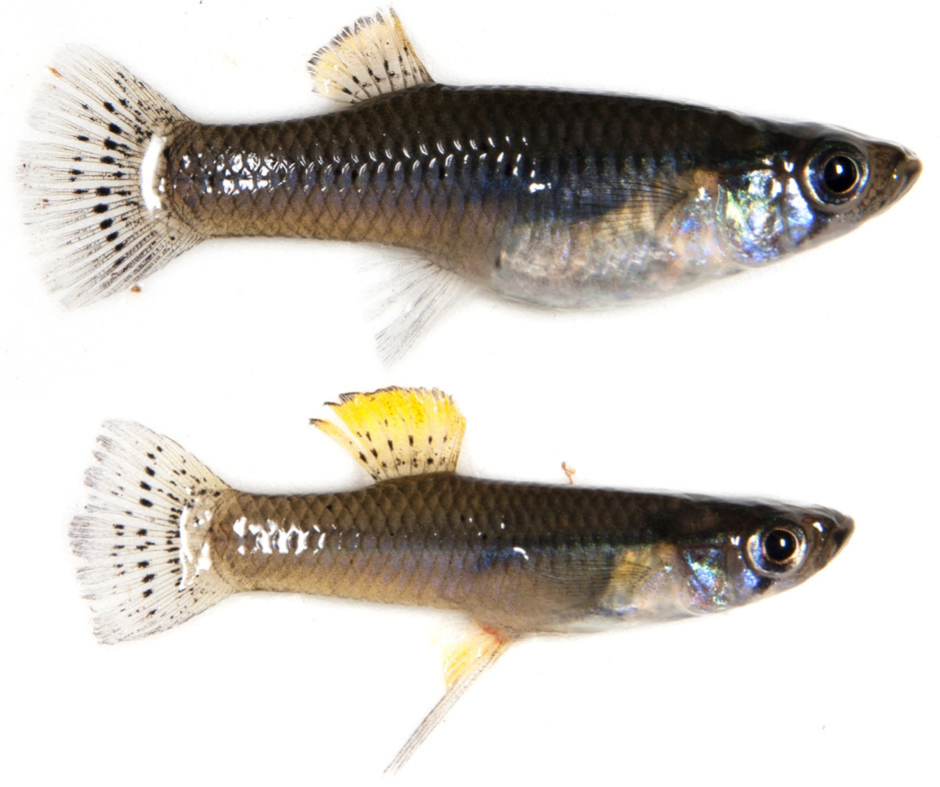
Female (top) and male (bottom) *Gambusia hubbsi* inhabiting one of the studied tidal creeks in Abaco, Bahamas.

In the present study, we used the poeciliid fish *Gambusia hubbsi* (Fig. 1) as a model for understanding the consequences of human-induced ecosystem fragmentation on niche variation. Specifically, we used a path analysis approach to investigate factors influencing patterns of diet variation in *Gambusia*. We predicted that (i) habitat fragmentation should reduce the density of piscivores; (ii) as a consequence of predation release, *Gambusia* should increase in density and experience stronger intraspecific competition for food; and (iii) populations in fragmented areas should show more diet variation.

## Materials and Methods

### Study system

We examined wetlands on Abaco Island, Bahamas. These systems, locally called “tidal creeks,” are characterized by a relatively narrow creek mouth that provides the primary conduit for tidal exchange (semi-diurnal tidal regime, ∼0.8 meter tidal amplitude). Creeks typically broaden moving landward from the mouth, grading into expanses of shallow (<1 m at low tide) wetlands with red mangrove (*Rhizophora mangle*) as the primary emergent vegetation. These systems generally have small watersheds with little freshwater input, being dominated by marine waters with predictable tidal flow, and characterized by marine flora and fauna.

One of the most common forms of habitat alteration in coastal wetlands, including tidal creeks, is fragmentation. In the Bahamas, fragmentation typically results from roads constructed across a tidal creek, usually near the creek mouth. These roads greatly reduce hydrological connectivity, that is, the water-mediated transfer of matter, energy, or organisms within or between elements of the hydrological cycle (Pringle 2001, 2003a,b). In some cases, water-flow conveyance structures, such as culverts, mitigate these hydrological impacts. As a consequence, Bahamian tidal creeks show a broad gradient of fragmentation. At one extreme, natural, unfragmented tidal creeks exhibit high connectivity to adjacent marine areas. At the other extreme, wetlands are completely fragmented, resulting in isolated systems with no connectivity. The dramatic changes in biotic characteristics of fragmented tidal creeks include an overall reduction in the abundance of marine piscivores, fewer basal resource pools, and a simplification of food web structure (Layman et al. 2004; Valentine-Rose et al. 2007a,b; Valentine-Rose and Layman 2011; Valentine-Rose et al. 2011; Table A1 in the Appendix S1).

### Data collection

We surveyed 13 tidal creeks across the gradient of fragmentation (Table A2 in the Appendix S2). We measured 10 variables (abiotic and structural aspects) at each site to capture features of tidal creeks that may be directly influenced by fragmentation (Table A3 in the Appendix S2). Measurements were made multiple times per year, and we used the mean of annual averages based on 3–6 years of sampling at these sites (between 2006–2012), with the exception of distance to creek mouth, ecosystem size, turbidity, and mangrove perimeter which were measured once in 2010.

#### Relative piscivore and *Gambusia* densities

We quantified the abundance of piscivorous fishes (Table A1 in the Appendix S1) and *Gambusia* in the study sites. We estimated piscivore density between three and seven times per site between 2009 and 2010 and *Gambusia* density during two separate surveys for 12 of the 13 sites (July 2009 and March 2010). We found significant repeatability in both density estimates [piscivore intraclass correlation coefficient: *r* = 0.83, *P* < 0.0001; *Gambusia*: *r* = 0.72, *P* = 0.0025 (following Lessells and Boag 1987)]. This indicates that our density estimates confer a reasonable level of confidence for comparing relative densities across sites. Piscivore abundances were estimated with underwater visual census (Nagelkerken et al. 2000; Layman et al. 2004), which provides relative estimates of predator abundances in Bahamian tidal creeks (Valentine-Rose et al. 2007b). *Gambusia* densities were estimated visually using quadrats of 0.25 m^2^ area. Further details on these methods are given in the Appendix S3.

#### Growth rates

Changes in the densities of *Gambusia* can potentially lead to changes in the degree of intraspecific competition for food. Higher intraspecific competition is expected to depress individual growth rates (Svanbäck and Bolnick 2007). In order to evaluate this possibility, we measured the ratio of RNA-to-DNA concentration (RNA/DNA) as a proxy for growth rate. Faster growing fish synthesize more proteins and hence have a higher RNA titer per cell, whereas the concentration of DNA in cells is constant through time (Dahlhoff 2004). As a consequence, RNA/DNA has been shown to be tightly correlated with growth rate in several fishes (Caldarone et al. 2001; Ali and Wootton 2003; Dahlhoff 2004).

In 2009, 24–91 fish were collected with dip nets from each of 11 populations (Table A4 in the Appendix S4). Upon collection, individuals were euthanized in eugenol, and a ∼5 mg sample of muscle tissue was immediately removed from the caudal peduncle and preserved in RNAlater (Ambion®; Life Technologies, Austin, TX). Tissue samples were refrigerated for ∼24 h and then frozen at −20°C until RNA/DNA quantitation. We quantified RNA/DNA in muscle tissue, following Bolnick and Lau (2008). In the laboratory, individuals were weighed (0.01 g) and dissected. Upon dissection, age class (juvenile vs. adult) and sex were determined by gonad inspection. To ensure that our standardized tissue-removal procedure did not introduce any bias to our estimates of body size, we also calculated standard length (SL) for 445 fish in our dataset and examined the correlation of these two estimates of body size. We found very high log-log correlation between mass and SL (*r* = 0.98, *P* < 0.0001), indicating that our weight measurements provided unbiased estimates of body size for comparison among individuals.

#### Gut contents and stable isotopes

In 2009, we performed gut content analysis in three representative sites to obtain preliminary patterns and assess power for detection of interindividual diet variation within sites. The sites chosen were Sand Bar (*n* = 35 individuals), Sandy Point (*n* = 64), and the upstream portion of Double Blocked (*n* = 48). We chose these sites because they are representative of an unaltered, moderately connected, and totally fragmented area, respectively, spanning the range of fragmentation of Bahamian estuaries (Table A2 in the Appendix S2). As indicated by simulations, we had power to accurately estimate the degree of interindividual diet variation with samples as small as 12 individuals (Fig. A1 in the Appendix S5). Using these results as a guide, we then collected fish in 2010 from 13 tidal creeks across the connectivity gradient, examining 12 individuals from each site (Table A4 in the Appendix S4), providing for our primary investigation of the effects of fragmentation on patterns of diet variation. For the sake of comparisons among populations, we used individuals from the same age class and sex (adult females). Specimens were immediately euthanized and preserved in 95% ethanol upon collection. In the laboratory, individuals were dissected for removal of guts. Gut contents were analyzed under a stereo microscope. Prey items were counted and identified to the lowest feasible taxonomic level.

We complemented gut content analysis with stable isotopes, which reflect longer term trophic relationships. Because the variation in isotope values among individuals of a population provides a measure of variation among their diets (Araújo et al. 2007), stable isotopes can be a useful tool in measuring interindividual diet variation that can be used in conjunction with gut content analysis (Layman et al. 2012). We analyzed *δ*^13^C and *δ*^15^N stable isotope values of 14–64 fish from each of 10 populations (Table A4 in the Appendix S4). For stable isotope analysis, specimens were frozen upon collection. We measured stable isotopes of the whole fish after removing their digestive tract by dissection. Fish were dried, ground to a fine powder, encapsulated, and analyzed at the Yale Earth System Center for Stable Isotopic Studies (ESCSIS).

### Data analysis

#### Growth rates

We calculated population means for growth rate (RNA/DNA) separately for each age class/sex because RNA/DNA differed among males, females, and juveniles. For females and juveniles, growth rate was body size dependent (significant positive association with log mass; *P* < 0.001 in both cases), and thus, we calculated marginal means from a general linear model including log mass as a covariate to control for body size (for females we additionally included the interaction between population and log mass because there was some variation in the strength of the relationship among populations; results are qualitatively similar if excluding this interaction). For males, there was no association between body size and growth rate (*P* = 0.89), and thus, site means were used in analysis.

#### Gut contents and stable isotopes

Resource use was quantified as proportions based on the number of diet items found in gut contents. We quantified the diversity of resources consumed by each population (niche width) and the extent to which individuals are specialized in relation to the population. We measured the population niche width with Roughgarden's (1979) total niche width (TNW), which is Shannon's diversity index applied to the population diet proportions obtained from gut content analysis. In order to quantify the degree of individual specialization, we used the IS index (Bolnick et al. 2002), which is the average overlap between each individual's niche and the population niche, being 1 in the absence of individual specialization (individuals overlap completely with the population) and assuming lower values as individuals’ niches become smaller subsets of the population niche (higher individual specialization). In order to make this index more intuitive, we used *V* = 1 − IS, so that in the absence of individual specialization *V* equals zero, assuming higher decimal values as individuals become more specialized (Bolnick et al. 2007).

We used a Bayesian approach based on multivariate ellipse-based metrics of the stable isotope data as an estimate of diet variation (Jackson et al. 2011). This approach is especially useful when comparing populations with different sample sizes (Layman et al. 2012). The analysis generates standard ellipse areas (SEA), which are bivariate equivalents to standard deviations in univariate analysis. Larger areas correspond to a more diverse isotopic niche, that is, a larger proportion of isotopic niche space occupied (in this case, bivariate *δ*^13^C or *δ*^15^N space) because of more variation among individuals.

#### Effects of habitat fragmentation

**Figure 2:**
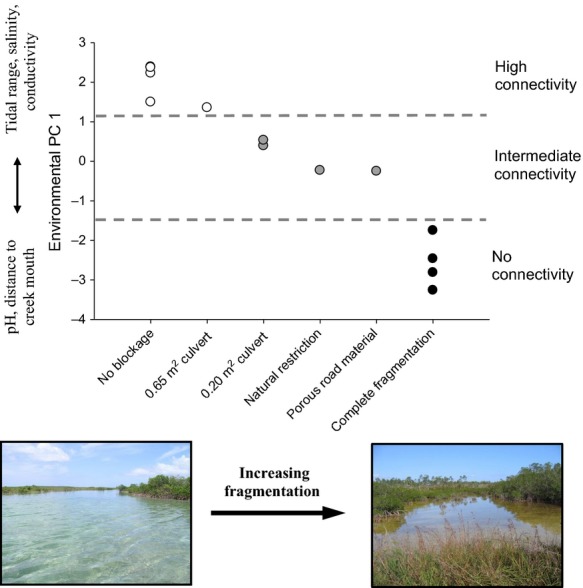
Relationship between environmental variation and degree of fragmentation of 13 tidal creeks in Abaco, Bahamas. Sites were ordered by PC1-scores and a brief description of their fragmentation status is given on the *x*-axis. Overall, PC1-scores map onto the degree of fragmentation. See Table A2 for details on the sites.

To summarize environmental variation among sites in abiotic and structural features, we conducted a principal components analysis (using the correlation matrix) with the 10 variables measured for each site. We retained the first four PC axes, accounting for more than 90% of the variance (Table 1). We found that PC1 exhibited a direct and clear association with degree of fragmentation (Fig. 2), but other PCs exhibited no such relationship (not presented). PC1 captured much of the variance in the data, with positive scores associated with higher salinities, greater tidal fluctuations, greater conductivity, and (more weakly with) lower pH and a shorter distance to the creek mouth. Altogether, our four PCs summarized environmental variation both associated with, and independent of, ecosystem fragmentation. We used these four PCs in analyses described below.

**Table 1:** Results of principal components analysis of the 10 environmental variables measured at 13 tidal creek systems in the Bahamas

Variable	PC1	PC2	PC3	PC4
Mean tidal range (m)	**0.83**	0.44	0.28	0.14
Maximum tidal range (m)	**0.86**	0.38	0.28	0.13
Distance to creek mouth (m)	−**0.66**	0.35	0.16	−0.45
Ecosystem size (m^2^)	0.24	**0.63**	**0.64**	−0.24
Maximum water depth (cm)	−0.32	0.44	−0.14	**0.75**
pH	−**0.71**	0.20	0.54	0.21
Salinity (ppt)	**0.90**	−0.31	0.09	−0.01
Conductivity (mS)	**0.79**	−0.45	−0.07	−0.04
Turbidity (NTU)	−0.17	−**0.71**	0.49	0.40
Mangrove perimeter (%)	0.25	**0.76**	−0.51	0.11
Percentage of variance	40.50	24.81	14.09	10.66

PC loadings ≥ |0.6| in bold type.

We used path analysis (e.g., Kline 2005) to investigate direct and indirect effects of environmental variables (4 PCs) on piscivore and *Gambusia* densities (log_10_-transformed) and *Gambusia* diet and growth rate. We constructed a full path diagram based on hypotheses regarding how environmental variation might lead to diet variation in *Gambusia*. Our full path diagram included potential pathways leading from: (i) all four environmental PCs to piscivore density, (ii) all four environmental PCs and piscivore density to *Gambusia* density, and (iii) all four environmental PCs, and either piscivore density or *Gambusia* density (but not both) to each diet and growth rate variable. We did not include paths from both piscivore and *Gambusia* density simultaneously because of multicollinearity among these variables (Variance Inflation Factors up to 12.09 in those cases; VIFs > 10 are typically considered problematic, for example, Myers 1990). We employed model selection using Akaike information criterion corrected for small sample sizes (AIC_*c*_; Akaike 1992; Burnham and Anderson 2002) to select the best subset of paths leading to each endogenous variable. Multicollinearity was generally low in considered models (all VIFs < 10), with the highest VIF in the final analyses being 1.23.

All path coefficients were calculated as standardized (partial) regression coefficients estimated using 1000 bootstraps of the dataset. We assessed significance of direct effects (path coefficients) and total effects (sum of direct and indirect effects) using a bootstrap approximation obtained by constructing two-sided bootstrapped confidence intervals. These bootstrapping approaches provide more accurate estimates of path coefficients and their errors for datasets with relatively small sample sizes (Bollen and Stine 1990; MacKinnon et al. 2004). Path analysis was conducted with Amos version 18 (Arbuckle 2003). We conducted the path analysis in multiple steps due to varying sample sizes for some variables (i.e., stable isotopes and RNA/DNA data were not available for all sites), and because growth rate data was analyzed separately for each age-sex category.

## Results

**Figure 3:**
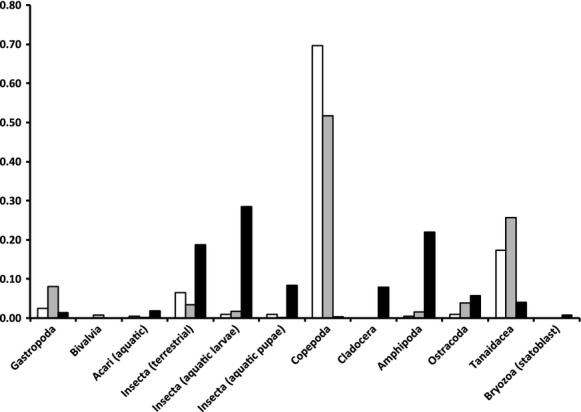
Diet composition of the 13 analyzed populations. Populations were pooled together according to level of connectivity (colors follow Figure 2). Diets are represented as the proportions of the number of prey items consumed.

We found that habitat fragmentation of Bahamian tidal creeks caused a sharp decrease in piscivore and an increase in *Gambusia* densities (Table 2). This trend was accompanied by an overall decrease in growth rates in fragmented areas. Diet variation tended to be higher in fragmented areas, which was associated with a shift from a diet mainly composed of copepods to the inclusion of additional aquatic and allochthonous invertebrates (Fig. 3).

**Table 2:** Number of piscivores, *Gambusia* densities, RNA/DNA ratios (females/males/juveniles), total niche width (TNW), the *V* index of individual specialization, *δ*^13^C and *δ*^15^N [mean (SD)], and stable isotope standard ellipse areas (SEA) of the 13 studied areas

Site	Connectivity	Piscivores (ind/0.3 ha)	*Gambusia* (ind/m^2^)	RNA/DNA	TNW	V	*δ*^13^C	*δ*^15^N	SEA
Sand Bar	High	316	0	2.94/1.45/2.45	1.27	0.52	−17.6 (1.15)	6.5 (0.39)	1.34
Twisted Bridge	High	365	0	1.90/0.92/1.41	0.61	0.16	−15.22 (0.64)	7.1 (0.37)	0.70
Cherokee Sound	High	56	0.3	2.75/1.19/1.92	0.86	0.20	−17.29 (0.92)	6.8 (0.52)	1.23
Blue Holes	High	118	0.3	1.65/1.17/1.70	1.02	0.33			
Treasure Cay	High	78	0.2	1.49/1.05/1.53	1.49	0.40	−18.9 (0.75)	7.3 (0.46)	1.05
Crossing Rocks	Intermediate	40	7.6	1.34/0.80/1.20	1.88	0.60	−15.0 (1.07)	8.3 (0.43)	1.54
Sandy Point	Intermediate	1	15.8	1.35/1.06/1.59	1.23	0.43	−16.8 (1.31)	6.5 (0.41)	1.73
Indian River West	Intermediate	163	2.3	1.42/0.92/1.53	1.55	0.50	−22.5 (0.79)	9.3 (0.19)	0.51
Loggerhead Creek	Intermediate	4	7.4		1.27	0.52			
Indian River East	None	0	12.8		1.66	0.60			
Stinky Pond	None	0	5.1	1.98/1.20/1.38	1.35	0.38	−23.0 (2.55)	7.6 (0.95)	7.57
Double Blocked-Down	None	10	4.5	1.59/1.28/1.31	0.97	0.44	−23.1 (0.53)	8.1 (0.49)	0.83
Double Blocked-Up	None	0	10.7	0.16/0.51/0.66	1.81	0.55	−25.7 (2.35)	8.1 (0.65)	4.91

For males we present RNA/DNA site means; for females and juveniles we present marginal means from a general linear model including log mass as a covariate.

We selected the best subset of paths for our path analysis based on AIC_*c*_, which resulted in a total of nine direct paths out of a total of 30 possible paths. In many cases, the best model was selected unambiguously, but in some cases multiple models exhibited similar AIC_*c*_ values (within 2 AIC_*c*_ units; Appendix S6). In all cases, we selected the top model. Only three cases resulted in highly ambiguous results (pathways to male RNA/DNA, SEA, and *Gambusia* density), and in every case the next-best model was a subset of the best model—we chose the more complex model as we wished to identify any environmental factor that might play an important explanatory role.

**Figure 4:**
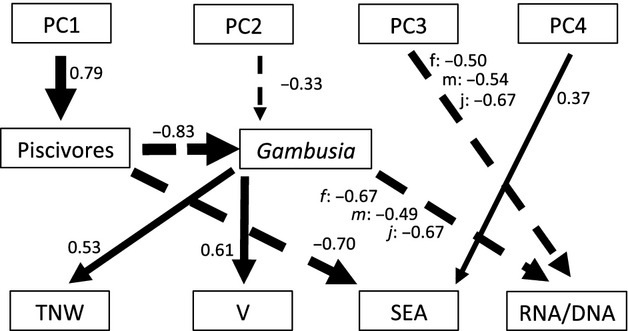
Path analysis results. Numerical values indicate standardized path coefficients, and line thickness reflects the strength of the path. Solid lines represent positive effects, and dashed lines represent negative effects. f: females; m: males; j: juveniles. total niche width (TNW), V, and standard ellipse areas (SEA) as in Table 2.

The resulting path analysis identified many strong relationships, with all paths but one being statistically significant (Fig. 4, Table 3). Examination of total effects of all factors revealed many indirect effects in addition to direct effects (Table 3). The most ubiquitous factor exhibiting significant total effects was environmental PC1, which described features strongly associated with tidal-creek fragmentation (Fig. 2). This variable was significantly associated with every endogenous variable in the path analysis, in line with our *a priori* predictions of the effects of habitat fragmentation of Bahamian estuaries on piscivore and *Gambusia* densities and its consequences on *Gambusia* growth rates and diet variation (Table 3). The primary results of our path analysis can be summarized as follows: (i) habitat connectivity has a positive effect on the density of piscivores; (ii) density of piscivores in turn has a negative effect on the density of *Gambusia*; (iii) *Gambusia* density has a negative association with growth rate in all age-sex categories; (iv) *Gambusia* density has a positive association with diet variation; and (v) stable isotope variation is more influenced by predators than *Gambusia* density. We also identified other environmental factors important in affecting *Gambusia* diet variation, such as ecosystem size, water pH, turbidity, and surrounding mangrove habitat. Importantly, the proximate mechanisms of *Gambusia* and predator densities provided better explanations than hydrological connectivity per se, including unmeasured factors co-varying with connectivity (i.e., no direct effects of PC1 on diet or growth variables). In summary, results indicate that more highly fragmented sites tended to have reduced piscivore density, increased *Gambusia* density, reduced growth rates, and increased trophic niche diversity.

**Table 3:** Summary of total effects (combined direct and indirect effects) revealed by path analysis

	PC1		PC2		PC3		PC4		Piscivore density		*Gambusia* density	
						
Effect on	*β*	*P*	*β*	*P*	*β*	*P*	*β*	*P*	*β*	*P*	*β*	*P*
Piscivore density	0.79	0.004										
*Gambusia* density	−0.65	0.007	−0.33	0.046					−0.83	0.009		
Total niche width (TNW)	−0.35	0.018	−0.18	0.025					−0.44	0.017	0.53	0.018
Individual specialization (V)	−0.40	0.015	−0.20	0.029					−0.50	0.02	0.61	0.018
Isotopic Ellipse Area (SEA)	−0.55	0.025					0.37	0.173	−0.70	0.012		
Female growth rate (RD)	0.44	0.003	0.22	0.005	−0.50	0.003			0.55	0.003	−0.67	0.002
Male growth rate (RD)	0.32	0.011	0.16	0.007	−0.54	0.016			0.40	0.012	−0.49	0.006
Juvenile growth rate (RD)	0.43	0.002	0.22	0.007	−0.56	0.001			0.55	0.003	−0.67	0.002

## Discussion

In the present study, we found evidence that habitat fragmentation of Bahamian tidal creeks results in loss of piscivorous fish, likely allowing for increased population densities of *Gambusia* and higher levels of intraspecific competition for food. As a result, populations in fragmented areas show a broadening of their food niche, which is achieved via increased interindividual diet variation. In the following paragraphs, we elaborate on the mechanisms driving this pattern and its potential ecological and evolutionary consequences.

Environmental impacts of human-induced ecosystem fragmentation in Bahamian tidal creeks were captured by a single PC in our dataset (see Fig. 2) describing the dramatic, direct consequences of fragmentation. Our path analysis revealed that habitat fragmentation (PC1) had a strong direct effect on a single measured variable only, piscivore density, but that this effect cascaded to produce significant indirect effects on all measured aspects of *Gambusia* populations. This suggests that predatory release provides the primary mechanism underlying changes in the population ecology of *Gambusia* following fragmentation.

We found strong evidence that predatory release in more fragmented localities resulted in increased *Gambusia* densities—a finding consistent with prior work in other poeciliid fish systems demonstrating higher densities and lower mortality rates in sites with lower predator densities (Reznick and Bryant 2007; Johnson and Zúñiga-Vega 2009; Heinen et al. 2013). Alternatively, increased *Gambusia* densities might result from greater resource productivity (unmeasured in this study), which happened to negatively co-vary with piscivore density. We find this explanation unlikely, as such co-variation is not known or expected to exist, growth rates of *Gambusia* were lower, not higher, in fragmented sites, and previous work on *Gambusia* inhabiting blue holes on Andros Island, Bahamas has demonstrated much higher densities in the absence of piscivorous fish and no relationship between *Gambusia* density and measures of resource productivity (Heinen et al. 2013).

The observed trend of higher trophic niche diversity in fragmented areas could be explained by three different primary mechanisms. First, the underlying cause might not have been captured by our path analysis, in which increased diet diversity resulted from increased diversity of the resource base available to *Gambusia* following fragmentation. Available studies, however, point to a general simplification of food webs and energy-flow pathways, as well as a reduction in species richness, in Bahamian fragmented estuaries (Layman et al. 2007, 2010; Valentine-Rose et al. 2007a). Therefore, we find this mechanism unlikely, although we acknowledge that we cannot definitively rule it out without the quantification of resource pools available to *Gambusia* across the fragmentation gradient.

Second, predatory release in fragmented areas, resulting in increased *Gambusia* densities, can result in elevated diet variation via increased levels of intraspecific competition. Our results strongly support this notion, as sites with greater *Gambusia* densities experienced (i) reduced growth rates, (ii) increased population niche width, and (iii) increased individual diet specialization. Our findings, therefore, add to the growing literature, suggesting a positive effect of intraspecific competition on diet variation (reviewed in Araújo et al. 2011). It is not clear how interspecific competition, which is also expected to affect the degree of individual specialization (Bolnick et al. 2010), should change with fragmentation and impact *Gambusia*, but available data suggest it should generally be weak. Redear herring (*Harengula humeralis*) and hardhead silversides (*Atherinomorus stipes*), which are planktivores and possible competitors, have rather low densities in most sites and show reduced densities in fragmented areas (Table A1 in the Appendix S1); sheepshead minnows (*Cyprinodon variegatus*) increase in abundance in fragmented areas, but show relatively little dietary and microhabitat overlap with *Gambusia* (see Martin and Wainwright 2011, 2013).

Finally, increased diet diversity could result from behavioral changes in *Gambusia*, rather than density-mediated effects of predatory release. Specifically, predators can alter diet patterns of prey species via predator-induced prey behaviors, such as refuge use or reduced overall activity (Werner et al. 1983; Eklöv and Svanbäck 2006). We found support for this mechanism in the present study from the strong direct effect of piscivore density—not *Gambusia* density—on isotopic variation. This finding could reflect behavioral changes in *Gambusia* associated with predation intensity, such as shifts in habitat use or shoaling behavior. Indeed, observations during this study, and previous work on *Gambusia* in both tidal creeks and blue holes, suggest that *Gambusia* individuals remain relatively restricted to shallow, near-shore, regions in high-predation localities, but exploit offshore waters in low-predation sites where they utilize much more of the available water column (Heinen et al. 2013). Moreover, recent experimental work in a congener, *G. affinis*, demonstrated that reduced shoaling intensity, as occurred in the absence of predators, led to increased diet diversity and specialization (C. Filla, A. M. Makowicz, R. B. Langerhans, unpubl. manuscript). Thus, more dispersed habitat use (reduced shoaling, offshore use) in fragmented sites with reduced piscivore density might have led to the opportunity to feed on prey that may have different *δ*^13^C and *δ*^15^N signatures. This result suggests that predator-induced behavioral changes manifest as longer term dietary effects (captured by stable isotopes), whereas recent feeding patterns (revealed by gut contents) are better predicted by *Gambusia* densities. Thus, our results suggest that both density-mediated and behavior-mediated effects of predatory release, subsequent to habitat fragmentation, may be responsible for driving changes in patterns of diet diversity and specialization in *Gambusia*.

We acknowledge that the variation among consumers’ stable isotopes will not only depend on diet variation but also on the variation among isotope baselines, which we did not quantify. However, fragmentation of Bahamian tidal creeks results in a dramatic reduction of the diversity of basal resource pools, which is reflected in smaller ranges of *δ*^13^C of food webs in fragmented areas (Layman et al. 2007). Therefore, the variation in baselines among sites is unlikely to explain the trend of higher isotope variation in *Gambusia* in more fragmented areas. This trend, which corresponds closely with gut content data, is likely a reflection of actual diet variation.

Might the observed patterns of resource use in *Gambusia* have ecosystem-level consequences? Previous experimental studies on the Trinidadian guppy *Poecilia reticulata* have shown that differences in the diets of populations inhabiting low- versus high-predation environments can substantially change ecosystem structure and function – such as standing stocks of producers and consumers, as well as primary productivity and nutrient flow (Palkovacs et al. 2009; Bassar et al. 2010; Marshall et al. 2012). Therefore, the changes in resource use and population densities of *Gambusia* associated with habitat fragmentation observed in this study can potentially impact important ecological aspects of Bahamian estuarine ecosystems in ways that are currently not known.

Another implication of our findings concerns the potential local adaptation of *Gambusia* populations, with implications for evolutionary diversification (Langerhans et al. 2007). Several poeciliid species are known to experience divergent natural selection between predation regimes on body morphology, body color, life histories, and physiological performance capacities, with subsequent evolutionary divergence, and even speciation, in some systems (Langerhans et al. 2007; Reznick et al. 2008; Langerhans 2010; Heinen et al. 2013; Riesch et al. 2013; Martin et al. 2014). By creating novel predator-free environments, habitat fragmentation may drive divergent selection on multiple *Gambusia* phenotypes, and this study suggests such selection may additionally relate to trophic morphology due to divergent diets. Ecologists are becoming increasingly aware that populations may show rapid evolutionary responses to habitat degradation (Smith and Bernatchez 2008; De León et al. 2011; Franssen 2011; Sih et al. 2011). The study system examined here appears ripe for investigation of contemporary evolution driven by human activities, as fragmentation leads to strong ecological differences with known selective effects, and fragmentation of wetlands via road construction is widespread.

## Supporting Information

**Appendix S1**. List of potential piscivorous predators and competitors of *G. hubbsi*. **Appendix S2**. Characterization of the studied sites. **Appendix S3**. Details on the methods used to estimate relative abundances of piscivorous fishes and *G. hubbsi* across the studied areas. **Appendix S4**. Sample sizes used in RNA/DNA, stable isotope, and gut content analyses. **Appendix S5**. Determination of sample sizes required to accurately estimate the IS index of individual specialization. **Appendix S6**. Model Selection Results.

## References

[b1] Akaike H (1992). Information theory and an extension of the maximum likelihood principle. Breakthroughs in statistics.

[b2] Ali M, Wootton RJ (2003). Correlates of growth in juvenile three-spined sticklebacks: potential predictors of growth rates in natural populations. Ecol. Freshw. Fish.

[b3] Araújo MS, Bolnick DI, Machado G, Giaretta AA, Reis SF (2007). Using d^13^C stable isotopes to quantify individual-level diet variation. Oecologia.

[b4] Araújo MS, Bolnick DI, Layman CA (2011). The ecological causes of individual specialisation. Ecol. Lett.

[b5] Arbuckle JL (2003).

[b6] Bassar RD, Marshall MC, Lopez-Sepulcre A, Zandona E, Auer SK, Travis J (2010). Local adaptation in Trinidadian guppies alters ecosystem processes. Proc. Natl Acad. Sci. USA.

[b7] Bollen KA, Stine RA (1990). Direct and indirect effects: classical and bootstrap estimates of variability. Sociol. Methodol.

[b8] Bolnick DI, Lau OL (2008). Predictable patterns of disruptive selection in stickleback in postglacial lakes. Am. Nat.

[b9] Bolnick DI, Yang LH, Fordyce JA, Davis JM, Svanbäck R (2002). Measuring individual-level resource specialization. Ecology.

[b10] Bolnick DI, Svanbäck R, Fordyce JA, Yang LH, Davis JM, Hulsey CD (2003). The ecology of individuals: incidence and implications of individual specialization. Am. Nat.

[b11] Bolnick DI, Svanbäck R, Araújo MS, Persson L (2007). Comparative support for the niche variation hypothesis that more generalized populations are also more heterogeneous. Proc. Natl. Acad. Sci. U.S.A.

[b12] Bolnick DI, Ingram T, Stutz WE, Snowberg LK, Lau OL, Paull JS (2010). Ecological release from interspecific competition leads to decoupled changes in population and individual niche width. Proc. Biol. Sci.

[b13] Bolnick DI, Amarasekare P, Araújo MS, Bürger R, Levine J, Novak M (2011). Why intraspecific trait variation matters in community ecology. Trends Ecol. Evol.

[b14] Burnham KP, Anderson DR (2002). Model selection and multi model inference: a practical information-theoretic approach.

[b15] Caldarone EM, Wagner M, Ogner-Burns JS, Buckley LJ (2001). Protocol and guide for estimating nucleic acids in larval fish using a fluorescence microplate reader.

[b16] Dahlhoff EP (2004). Biochemical indicators of stress and metabolism: applications for marine ecological studies. Annu. Rev. Physiol.

[b17] De León LF, Raeymaekers JAM, Bermingham E, Podos J, Herrel A, Hendry AP (2011). Exploring possible human influences on the evolution of Darwin's finches. Evolution.

[b18] Debinski DM, Holt RD (2000). A survey and overview of habitat fragmentation experiments. Conserv. Biol.

[b19] Dirzo R, Raven PH (2003). Global state of biodiversity and loss. Annu. Rev. Environ. Resour.

[b20] Eklöv P, Svanbäck R (2006). Predation risk influences adaptive morphological variation in fish populations. Am. Nat.

[b21] Estes JA, Terborgh J, Brashares JS, Power ME, Berger J, Bond WJ (2011). Trophic downgrading of planet earth. Science.

[b22] Fahrig L (2003). Effects of habitat fragmentation on biodiversity. Annu. Rev. Ecol. Evol. Syst.

[b23] Fischer J, Lindenmayer DB (2007). Landscape modification and habitat fragmentation: a synthesis. Glob. Ecol. Biogeogr.

[b24] Foley JA, DeFries R, Asner GP, Barford C, Bonan G, Carpenter SR (2005). Global consequences of land use. Science.

[b25] Franssen NR (2011). Anthropogenic habitat alteration induces rapid morphological divergence in a native stream fish. Evol. Appl.

[b26] Harrison S, Bruna E (1999). Habitat fragmentation and large-scale conservation: what do we know for sure?. Ecography.

[b27] Heinen J, Coco M, Marcuard M, White D, Peterson MN, Martin R (2013). Environmental drivers of demographics, habitat use, and behavior during a post-Pleistocene radiation of Bahamas mosquitofish (*Gambusia hubbsi*. Evol. Ecol.

[b28] Jackson AL, Inger R, Parnell AC, Bearhop S (2011). Comparing isotopic niche widths among and within communities: SIBER – Stable Isotope Bayesian Ellipses in R. J. Anim. Ecol.

[b29] Johnson JB, Zúñiga-Vega JJ (2009). Differential mortality drives life-history evolution and population dynamics in the fish *Brachyrhaphis rhabdophora*. Ecology.

[b30] Kline R (2005). Details of path analysis. Principles and practice of structural equation modeling.

[b31] Langerhans RB (2010). Predicting evolution with generalized models of divergent selection: a case study with poeciliid fish. Integr. Comp. Biol.

[b32] Langerhans RB, Gifford ME, Joseph EO (2007). Ecological speciation in *Gambusia* fishes. Evolution.

[b33] Layman CA, Arrington DA, Langerhans RB, Silliman BR (2004). Degree of fragmentation affects fish assemblage structure in Andros Island (Bahamas) estuaries. Carib. J. Sci.

[b34] Layman CA, Quattrochi JP, Peyer CM, Allgeier JE (2007). Niche width collapse in a resilient top predator following ecosystem fragmentation. Ecol. Lett.

[b35] Layman CA, Arrington DA, Kramer PA, Valentine-Rose L, Dahlgren CP (2010). Indicator taxa to assess anthropogenic impacts in Caribbean and Bahamas tidal creeks. Carib. J. Sci.

[b36] Layman CA, Araújo MS, Boucek R, Hammerschlag-Peyer CM, Harrison E, Jud ZR (2012). Applying stable isotopes to examine food-web structure: an overview of analytical tools. Biol. Rev.

[b37] Lessells C, Boag PT (1987). Unrepeatable repeatabilities: a common mistake. Auk.

[b38] MacKinnon DP, Lockwood CM, Williams J (2004). Confidence limits for the indirect effect: distribution of the product and resampling methods. Multivar. Behav. Res.

[b39] Marshall MC, Binderup AJ, Zandonà E, Goutte S, Bassar RD, El-Sabaawi RW (2012). Effects of consumer interactions on benthic resources and ecosystem processes in a Neotropical stream. PLoS One.

[b40] Martin CH, Wainwright PC (2011). Trophic novelty is linked to exceptional rates of morphological diversification in two adaptive radiations of *Cyprinodon* pupfish. Evolution.

[b41] Martin CH, Wainwright PC (2013). Multiple fitness peaks on the adaptive landscape drive adaptive radiation in the wild. Science.

[b42] Martin RA, Riesch R, Heinen-Kay JL, Langerhans RB (2014). Evolution of male coloration during a post-pleistocene radiation of Bahamas mosquitofish (*Gambusia hubbsi*. Evolution.

[b43] Myers RH (1990). Classical and modern regression with applications.

[b44] Nagelkerken I, Dorenbosch M, Verbeck WCEP, Cocheret de la Morinière E, van der Velde G (2000). Importance of shallow-water biotopes of a Caribbean bay for juvenile coral reef fishes: patterns in biotope association, community structure and spatial distribution. Mar. Ecol. Prog. Ser.

[b45] Nilsson C, Reidy CA, Dynesius M, Revenga C (2005). Fragmentation and flow regulation of the world's large river systems. Science.

[b46] Palkovacs EP, Marshall MC, Lamphere BA, Lynch BR, Weese DJ, Fraser DF (2009). Experimental evaluation of evolution and coevolution as agents of ecosystem change in Trinidadian streams. Philos. Trans. R. Soc. Lond., B, Biol. Sci.

[b47] Pringle CM (2001). Hydrologic connectivity and the management of biological reserves: a global perspective. Ecol. Appl.

[b48] Pringle C (2003a). The need for a more predictive understanding of hydrologic connectivity. Aquat. Conserv.

[b49] Pringle C (2003b). What is hydrologic connectivity and why is it ecologically important?. Hydrol. Process.

[b50] Pringle C, Crooks KR, Sanjayan M (2006). Hydrologic connectivity: a neglected dimension of conservation biology. Connectivity conservation.

[b51] Reznick D, Bryant M (2007). Comparative long-term mark-recapture studies of guppies (*Poecilia reticulata*): differences among high and low predation localities in growth and survival. Ann. Zool. Fenn.

[b52] Reznick DN, Ghalambor CK, Crooks K (2008). Experimental studies of evolution in guppies: a model for understanding the evolutionary consequences of predator removal in natural communities. Mol. Ecol.

[b53] Riesch R, Martin RA, Langerhans RB (2013). Predation's role in life-history evolution of a livebearing fish and a test of the Trexler-DeAngelis model of maternal provisioning. Am. Nat.

[b54] Roughgarden J (1979). Theory of population genetics and evolutionary ecology: an introduction.

[b55] Rypel AL, Layman CA (2008). Degree of aquatic ecosystem fragmentation predicts population characteristics of gray snapper (*Lutjanus griseus*) in Caribbean tidal creeks. Can. J. Fish. Aquat. Sci.

[b56] Saunders DA, Hobbs RJ, Margules CR (1991). Biological consequences of ecosystem fragmentation: a review. Conserv. Biol.

[b57] Sih A, Ferrari MCO, Harris DJ (2011). Evolution and behavioural responses to human-induced rapid environmental change. Evol. Appl.

[b58] Smith TB, Bernatchez L (2008). Evolutionary change in human-altered environments. Mol. Ecol.

[b59] Stephens DW, Krebs JR (1986). Foraging theory.

[b60] Svanbäck R, Bolnick DI (2005). Intraspecific competition affects the strength of individual specialization: an optimal diet theory model. Evol. Ecol. Res.

[b61] Svanbäck R, Bolnick DI (2007). Intraspecific competition drives increased resource use diversity within a natural population. Proc. R. Soc. Lond. B Biol. Sci.

[b62] Turner IM (1996). Species loss in fragments of tropical rain forest: a review of the evidence. J. Appl. Ecol.

[b63] Valentine-Rose L, Layman CA (2011). Response of fish assemblage structure and function following restoration of two small Bahamian tidal creeks. Restor. Ecol.

[b64] Valentine-Rose L, Cherry JA, Culp JJ, Perez KE, Pollock JB, Arrington DA (2007a). Floral and faunal differences between fragmented and unfragmented Bahamian tidal creeks. Wetlands.

[b65] Valentine-Rose L, Layman CA, Arrington DA, Rypel AL (2007b). Habitat fragmentation decreases fish secondary production in Bahamian tidal creeks. Bull. Mar. Sci.

[b66] Valentine-Rose L, Rypel AL, Layman CA (2011). Community secondary production as a measure of ecosystem function: a case study with aquatic ecosystem fragmentation. Bull. Mar. Sci.

[b67] Van Valen L (1965). Morphological variation and width of ecological niche. Am. Nat.

[b68] Vitousek PM, Mooney HA, Lubchenco J, Melillo JM (1997). Human domination of Earth's ecosystems. Science.

[b69] Walsh MR, Reznick DN (2009). Phenotypic diversification across an environmental gradient: a role for predators and resource availability on the evolution of life histories. Evolution.

[b70] Werner EE, Gilliam JF, Hall DJ, Mittelbach GG (1983). An experimental test of the effects of predation risk on habitat use in fish. Ecology.

