# Supplementary material for: Ecosystem fragmentation drives increased diet variation in an endemic livebearing fish of the Bahamas
Source: Ecol Evol. 2014 Jul 31;4(16):3298–308. doi: 10.1002/ece3.1140 (PMC4222216; doi:10.1002/ece3.1140)
Supplement: Supplementary file 1 [file ece30004-3298-sd1.docx]

**Supporting Information**

**Ecosystem fragmentation drives increased diet variation in an endemic livebearing fish of the Bahamas**

Márcio S. Araújo*, R. Brian Langerhans, Sean T. Giery, and Craig A. Layman

*Email: msaraujo@rc.unesp.br

**Appendix 1: List of potential piscivorous predators and competitors of *G. hubbsi*.**

**Appendix 2: Characterization of the studied sites.**

**Appendix 3: Details on the methods used to estimate relative abundances of piscivorous predators and *G. hubbsi* across the studied areas.**

**Appendix 4: Sample sizes used in RNA/DNA, stable isotope and gut content analyses.**

**Appendix 5: Determination of sample sizes required to accurately estimate the IS index of individual specialization.**

**Appendix 6: Model selection results.**

**Appendix 1**

**List of potential piscivorous predators and competitors of *G. hubbsi***

Table A1: Primary species of potential predators and competitors of Bahamas mosquitofish found in the studied-areas and the expected effect of habitat fragmentation on their abundances ([Layman et al. 2004](#_ENREF_2); [Valentine-Rose et al. 2007a](#_ENREF_4); [Valentine-Rose et al. 2007b](#_ENREF_6); [Valentine-Rose and Layman 2011](#_ENREF_5); [Valentine-Rose et al. 2011; unpubl. data](#_ENREF_7)).

| Scientific name | Common name | Effect of fragmentation on abundance |
| --- | --- | --- |
| Predatory fish |  |  |
| *Lutjanus griseus* | Gray snapper | Negative |
| *Lutjanus apodus* | Schoolmaster | Negative |
| *Lutjanus cyanopterus* | Cubera snapper | Negative |
| *Lutjanus jocu* | Dog snapper | Negative |
| *Sphyraena barracuda* | Great barracuda | Negative |
| *Strongylura notata* | Redfin needlefish | Negative |
| *Caranx latus* | Horse-eye jack | Negative |
| Potential competitor fishes |  |  |
| *Cyprinodon variegatus* | Sheepshead minnow | Positive |
| *Lophogobius cyprinoides* | Crested goby | Positive |
| *Gerres cinereus* | Yellow fin mojarra | Negative |
| *Eucinostomus* spp. | Mojarra | Negative |
| *Harengula humeralis* | Redear herring | Negative |
| *Atherinomorus stipes* | Hardhead silverside | Negative |

Layman, C. A., D. A. Arrington, R. B. Langerhans and B. R. Silliman (2004). "Degree of fragmentation affects fish assemblage structure in Andros Island (Bahamas) estuaries." Caribbean Journal of Science **40**: 232-244.

Valentine-Rose, L., J. A. Cherry, J. J. Culp, K. E. Perez, J. B. Pollock, D. A. Arrington and C. A. Layman (2007a). "Floral and faunal differences between fragmented and unfragmented Bahamian tidal creeks." Wetlands **27**: 702-718.

Valentine-Rose, L. and C. A. Layman (2011). "Response of fish assemblage structure and function following restoration of two small Bahamian tidal creeks." Restoration Ecology **19**(2): 205-215.

Valentine-Rose, L., C. A. Layman, D. A. Arrington and A. L. Rypel (2007b). "Habitat fragmentation decreases fish secondary production in Bahamian tidal creeks." Bulletin of Marine Science **80**: 863-877.

Valentine-Rose, L., A. Rypel and C. A. Layman (2011). "Community secondary production as a measure of ecosystem function: a case study with aquatic ecosystem fragmentation " Bulletin of Marine Science **87**(4): 913-937.

**Appendix 2**

**Characterization of the studied sites**

Table A2: Characterization of the degree of fragmentation of the 13 sites sampled. Sites are ordered from higher to lower PC1 scores (see Figure 2 in the main text).

| Site | Fragmentation status | Brief description |
| --- | --- | --- |
| Sand Bar | No blockage | Intertidal mangrove wetland |
| Twisted Bridge | No blockage | Intertidal mangrove wetland |
| Cherokee Sound | No blockage | Intertidal mangrove wetland |
| Blue Holes | No blockage | Small, rocky cove lined by mangroves |
| Treasure Cay | 0.65m^2^ culvert | Blocked by dirt road with a culvert allowing significant water flow. Piscivores move through culvert readily. |
| Crossing Rocks | 0.20m^2^ culvert | Shallow, rocky margin of an intertidal wetland blocked by a road with a culvert allowing water flow at high tide only. |
| Sandy Point | 0.20m^2^ culvert | Upper edge of an intertidal wetland blocked by a road with a culvert that allows water flow primarily at high tide. |
| Indian River-West side | Natural restriction | Deep, pond-like system that has a distant connection with the ocean. |
| Loggerhead Creek | Porous road material | Back rim of an intertidal wetland blocked by a road. Seepage through coarse rocks under the road provides some connectivity. |
| Indian River-East side | Complete fragmentation | Shallow mangrove wetland completely blocked by the main highway. |
| Stinky Pond | Complete fragmentation | Pond-like system with shallow wetland around the perimeter; periodically overwashed with marine waters in extreme storm events; a culvert runs under road, but typically is completely blocked with debris. |
| Double Blocked-Downstream | Complete fragmentation | Shallow, pond-like, system that is separated from marine waters by mangrove intrusion; some water exchange through mangrove roots at the highest tides. |
| Double Blocked-Upstream | Complete fragmentation | Shallow mangrove wetland completely blocked by a road. |

Table A3: Characterization of the 10 environmental variables measured at the 13 study sites.

| Site | Mean tidal range (m) | Maximum tidal range (m) | Distance to creek mouth (m) | Ecosystem size (m^2^) | Maximum water depth (cm) | pH | Salinity (ppt) | Conductivity (mS) | Turbidity (NTU) | Mangrove perimeter (%) |
| --- | --- | --- | --- | --- | --- | --- | --- | --- | --- | --- |
| Sand Bar | 0.51 | 0.67 | 120 | 100000 | 75 | 7.2 | 35.3 | 55.7 | 0.89 | 100 |
| Twisted Bridge | 0.88 | 1.13 | 130 | 500000 | 150 | 8.3 | 35.9 | 25.8 | 0.84 | 100 |
| Cherokee Sound | 0.50 | 0.75 | 340 | 350000 | 45 | 7.4 | 30.9 | 52.0 | 1.42 | 100 |
| Blue Holes | 0.53 | 0.73 | 1500 | 500000 | 200 | 7.5 | 31.7 | 46.9 | 1.90 | 70 |
| Treasure Cay | 0.46 | 0.64 | 1000 | 250000 | 180 | 8.1 | 35.0 | 54.7 | 1.01 | 100 |
| Crossing Rocks | 0.05 | 0.16 | 20 | 9000 | 40 | 7.3 | 35.6 | 42.8 | 2.77 | 40 |
| Sandy Point | 0.05 | 0.15 | 950 | 4000 | 40 | 7.7 | 36.2 | 56.8 | 1.30 | 60 |
| Indian River-West side | 0.26 | 0.39 | 1600 | 3000 | 220 | 7.7 | 15.4 | 41.4 | 1.10 | 100 |
| Loggerhead Creek | 0.16 | 0.31 | 150 | 1500 | 70 | 8.4 | 31.0 | 53.9 | 13.60 | 10 |
| Indian River-East side | 0.02 | 0.08 | 1150 | 110000 | 50 | 7.9 | 2.6 | 3.5 | 1.00 | 90 |
| Stinky Pond | 0.02 | 0.06 | 400 | 50000 | 450 | 8.4 | 4.1 | 3.8 | 3.34 | 90 |
| Double Blocked-Downstream | 0.01 | 0.03 | 3100 | 60000 | 180 | 8.7 | 7.2 | 12.7 | 2.08 | 95 |
| Double Blocked-Upstream | 0.01 | 0.05 | 3250 | 500000 | 100 | 8.9 | 1.4 | 0.3 | 2.36 | 40 |

Note: We used the mean of annual averages based on 3-6 years of sampling at these sites (between 2006-2012), with the exception of distance to creek mouth, ecosystem size, turbidity and mangrove perimeter which were measured once in 2010.

**Appendix 3**

**Details on the methods used to estimate relative abundances of piscivorous fishes and *G. hubbsi* across the studied areas**

We used underwater visual census ([UVC; Nagelkerken et al. 2000](#_ENREF_3); [Layman et al. 2004](#_ENREF_2)), which provides relative estimates of predator densities in Bahamas tidal creeks ([Valentine-Rose et al. 2007b](#_ENREF_6); [Valentine-Rose et al. 2011](#_ENREF_7)). In sites with water depths too shallow for UVC, we conducted predator surveys from above water by slowly walking the survey areas. In these shallow areas, all piscivores are readily visible from above water allowing for equally robust estimates of relative predator density. Visual surveys were conducted in an area of ~0.3ha in each site.

To estimate density of *G. hubbsi*, we performed surveys using 0.25m^2^ quadrats (n = 25-30/site). The quadrat was held ~0.5 m above the water to visualize the survey area. After 1 minute, the number of *G. hubbsi* swimming within the quadrat boundaries was recorded. To minimize disturbance to fish, the quadrat was held by a 1-m long handle. Piscivore density was estimated between three and seven times per site between 2009 and 2010 and *Gambusia* density during two separate surveys for 12 of the 13 sites (July 2009 and March 2010).

Valentine-Rose, L., C. A. Layman, D. A. Arrington and A. L. Rypel (2007b). "Habitat fragmentation decreases fish secondary production in Bahamian tidal creeks." Bulletin of Marine Science **80**: 863-877.

Valentine-Rose, L., A. Rypel and C. A. Layman (2011). "Community secondary production as a measure of ecosystem function: a case study with aquatic ecosystem fragmentation " Bulletin of Marine Science **87**(4): 913-937.

Layman, C. A., D. A. Arrington, R. B. Langerhans and B. R. Silliman (2004). "Degree of fragmentation affects fish assemblage structure in Andros Island (Bahamas) estuaries." Caribbean Journal of Science **40**: 232-244.

Nagelkerken, I., M. Dorenbosch, W. C. E. P. Verbeck, E. Cocheret de la Morinière and G. van der Velde (2000). "Importance of shallow-water biotopes of a Caribbean bay for juvenile coral reef fishes: patterns in biotope association, community structure and spatial distribution." Marine Ecology Progress Series **202**: 175-192.

**Appendix 4**

**Sample sizes used in RNA/DNA, stable isotope and gut content analyses**

Table A4: Number of specimens of *Gambusia hubbsi* analyzed for RNA/DNA, stable isotopes (SI), and gut contents in the comparison between 13 populations across a gradient of habitat fragmentation in Abaco, Bahamas. Populations were sampled in the summers of 2009 (RNA/DNA and SI) and 2010 (gut-contents).

| Site | RNA/DNA | SI | Gut contents* |
| --- | --- | --- | --- |
| Sand Bar | 46 | 35 | 12 |
| Twisted Bridge | 41 | 15 | 12 |
| Cherokee Sound | 42 | 15 | 12 |
| Blue Holes | 67 |  | 12 |
| Treasure Cay | 81 | 15 | 12 |
| Crossing Rocks | 40 | 16 | 12 |
| Sandy Point | 87 | 64 | 12 |
| Indian River-West side | 91 | 14 | 12 |
| Loggerhead Creek |  |  | 12 |
| Indian River-East side |  |  | 12 |
| Stinky Pond | 79 | 17 | 12 |
| Double Blocked-Downstream | 24 | 15 | 12 |
| Double Blocked-Upstream | 88 | 48 | 12 |

*Adult females.

**Appendix 5**

**Determination of sample sizes required to accurately estimate the IS index of individual specialization**

The representative sites chosen for this preliminary analysis were Sand Bar, Sandy Point, and the upstream area of Double Blocked (Double Blocked-Upstream; Table A2). We implemented a bootstrap procedure to evaluate the sample sizes required to obtain reliable estimates of the IS index of individual specialization ([Bolnick et al. 2002](#_ENREF_1)). IS represents the average degree of overlap between individuals' resource distributions and the overall population resource distribution. When individuals are ecologically identical, IS = 1, decreasing towards lower decimal values as individuals' niches become increasingly smaller subsets of the population niche and individuals are more heterogeneous. For each of the representative three populations, individuals from empirical samples were randomly sampled with replacement in order to build null populations of varying sizes (1-500 individuals), after which IS values were calculated. For each sample size, we calculated 1000 replicates, and then estimated average IS values and their standard errors. These randomizations indicated that it is possible to generate reliable estimates of IS in this system with as few as 12 fish per population, regardless of the degree of fragmentation of the site. This cut-off value guided examination of individual specialization across the 13 sites subsequently sampled. We would like to note that we were not interested in complete measures of diet variation, but instead relative estimates of variation across the fragmentation gradient. Any bias in estimation of diet variation within sites should be similar across all populations, since we standardized sample sizes. Simulations were implemented in C language, the codes of which are available upon request.

Bolnick, D. I., L. H. Yang, J. A. Fordyce, J. M. Davis and R. Svanbäck (2002). "Measuring individual-level resource specialization." Ecology **83**(10): 2936-2941.

Figure A1: Simulations testing the effect of sample size on the estimates of individual specialization (IS). Empirical samples from (a) Sand Bar (n = 35), (b) Sandy Point (n = 62), and (c) the upstream portion of Double Blocked-upstream (n = 47) were bootstrapped to generate null populations with sizes varying from 1 to 500 (only 1-30 shown). For each null population, IS was calculated. For each sample size, 1000 null populations were simulated, after which average values for the index (blue line) and standard errors (SE; red line) were calculated. Note rapid leveling off of estimates of IS and standard errors with small increases in sample size. Very minimal increases in parameter estimation occurred above a sample size of approximately 10-12 fish for any of the three sites.

**Appendix 6**

**Model Selection Results**

We only present up to the 10 best models in each case. Selected models are presented in **bold type**. Colors in figures represent levels of connectivity (following Fig. 2): high connectivity (blue), intermediate connectivity (yellow), and no connectivity (green).

**Log_10_ piscivore density:**

| Model | AIC*_c_* | ∆AIC*_c_* |
| --- | --- | --- |
| **PC1** | **30.87** | **0.00** |
| PC1 + PC2 | 32.40 | 1.53 |
| PC1 + PC4 | 35.13 | 4.26 |
| PC1 + PC3 | 35.18 | 4.31 |
| PC1 + PC2 + PC4 | 37.88 | 7.02 |
| PC1 + PC2 + PC3 | 37.94 | 7.08 |
| PC1 + PC3 + PC4 | 40.68 | 9.81 |
| PC2 | 43.17 | 12.30 |
| PC4 | 44.09 | 13.22 |
| PC3 | 44.11 | 13.24 |

**Log_10_ *Gambusia* density:**

| Model | AIC*_c_* | ∆AIC*_c_* |
| --- | --- | --- |
| **PC2 + log Piscivore density** | **6.47** | **0.00** |
| log Piscivore density | 7.32 | 0.85 |
| PC1 + PC2 | 8.22 | 1.74 |
| PC1 + PC2 + log Piscivore density | 8.66 | 2.18 |
| PC2 + PC4 + log Piscivore density | 9.47 | 3.00 |
| PC4 + log Piscivore density | 10.06 | 3.58 |
| PC1 + PC2 + PC4 | 10.50 | 4.02 |
| PC3 + log Piscivore density | 11.03 | 4.55 |
| PC2 + PC3 + log Piscivore density | 11.13 | 4.66 |
| PC1 + log Piscivore density | 11.33 | 4.86 |

**Total Niche Width (TNW):**

| Model | AIC*_c_* | ∆AIC*_c_* |
| --- | --- | --- |
| **log *Gambusia* density** | **14.08** | **0.00** |
| PC1 | 16.14 | 2.06 |
| log piscivore density | 16.70 | 2.63 |
| PC2 | 17.44 | 3.37 |
| PC3 + log *Gambusia* density | 17.70 | 3.63 |
| PC4 + log *Gambusia* density | 18.11 | 4.04 |
| PC2 + log *Gambusia* density | 18.32 | 4.25 |
| PC4 | 18.34 | 4.26 |
| PC3 | 18.34 | 4.26 |
| PC1 + PC2 | 18.34 | 4.26 |

**Individual Diet Specialization (V):**

| Model | AIC*_c_* | ∆AIC*_c_* |
| --- | --- | --- |
| **log *Gambusia* density** | **-13.66** | **0.00** |
| PC1 + PC2 | -11.98 | 1.68 |
| PC2 | -10.87 | 2.79 |
| PC2 + log *Gambusia* density | -10.54 | 3.12 |
| PC1 | -10.40 | 3.26 |
| PC3 + log *Gambusia* density | -10.32 | 3.35 |
| PC4 + log *Gambusia* density | -9.75 | 3.91 |
| PC1 + log *Gambusia* density | -9.36 | 4.31 |
| log piscivore density | -9.20 | 4.47 |
| PC1 + PC2 + PC4 | -8.56 | 5.10 |

**Standard Ellipse Area (SEA):**

| Model | AIC*_c_* | ∆AIC*_c_* |
| --- | --- | --- |
| **PC4 + log piscivore density** | **44.52** | **0.00** |
| log piscivore density | 45.32 | 0.80 |
| PC1 | 49.05 | 4.53 |
| PC2 + log piscivore density | 49.92 | 5.40 |
| PC1 + log piscivore density | 51.29 | 6.77 |
| PC3 + log piscivore density | 51.29 | 6.77 |
| PC4 | 51.75 | 7.23 |
| log *Gambusia* density | 51.75 | 7.23 |
| PC1 + PC4 | 51.93 | 7.41 |
| PC3 + PC4 + log piscivore density | 52.13 | 7.61 |

**Female RNA/DNA:**

| Model | AIC*_c_* | ∆AIC*_c_* |
| --- | --- | --- |
| **PC3 + log *Gambusia* density** | **24.77** | **0.00** |
| log *Gambusia* density | 26.91 | 2.14 |
| PC1 | 27.68 | 2.91 |
| PC2 + log *Gambusia* density | 29.54 | 4.77 |
| log piscivore density | 29.60 | 4.83 |
| PC1 + PC3 | 29.79 | 5.02 |
| PC1 + PC4 | 29.83 | 5.06 |
| PC3 | 30.96 | 6.19 |
| PC1 + log *Gambusia* density | 31.22 | 6.45 |
| PC4 + log *Gambusia* density | 31.36 | 6.59 |

**Male RNA/DNA:**

| Model | AIC*_c_* | ∆AIC*_c_* |
| --- | --- | --- |
| **PC3 + log *Gambusia* density** | **5.87** | **0.00** |
| PC3 | 6.30 | 0.43 |
| log *Gambusia* density | 7.05 | 1.18 |
| PC4 | 8.19 | 2.32 |
| PC1 | 8.38 | 2.51 |
| PC2 + PC3 | 8.53 | 2.66 |
| log piscivore density | 8.69 | 2.82 |
| PC2 | 9.60 | 3.72 |
| PC1 + PC3 | 10.06 | 4.19 |
| PC3 + log piscivore density | 10.38 | 4.51 |

**Juvenile RNA/DNA:**

| Model | AIC*_c_* | ∆AIC*_c_* |
| --- | --- | --- |
| **PC3 + log *Gambusia* density** | **12.15** | **0.00** |
| PC1 + PC3 | 13.61 | 1.45 |
| PC1 | 13.94 | 1.78 |
| PC2 + log *Gambusia* density | 15.37 | 3.21 |
| log *Gambusia* density | 16.11 | 3.96 |
| PC1 + PC3 + log *Gambusia* density | 16.72 | 4.57 |
| PC3 + PC4 + log *Gambusia* density | 17.04 | 4.88 |
| log piscivore density | 17.17 | 5.02 |
| PC2 + PC3 + log *Gambusia* density | 17.84 | 5.69 |
| PC1 + PC2 + PC3 | 17.85 | 5.70 |
